# Supplementary material for: Chromothripsis during telomere crisis is independent of NHEJ, and consistent with a replicative origin
Source: Genome Res. 2019 May;29(5):737–49. doi: 10.1101/gr.240705.118 (PMC6499312; doi:10.1101/gr.240705.118)
Supplement: Supplemental Material [file supp_gr.240705.118_Supplemental_file_1.zip › contigs/annotated_contigs/DB89/contig.2.DB89_length_633_mean_cov_6.3981042654.docx]

**DB89_length_633_mean_cov_6.3981042654**

```
ATCATTTTTATTAATTTTTAGTTTAATTTATTTTCAGCAAGATACGATGATGTGTATGATTTCCAACATTAAAAATTATTTATAAAGAC
 >chr18:63164304-63164584 - E=1e-153
CTTCTATATGGCACAACATATGGTCAACTTTGGTAAATGTCTAGTGCATACTTGAAAAGAAAGTACACTCTGCAGTTTAAGGAGTGCGA

TATCTTCACCATTAATTTTACTGATTGTGACGTCCTAATCTTTATTTTATCTTTACTGATTTTGGTGTCCTTGTTTATGAGTTTTTGAG

ATAGTTCGAATAA|AAAAAA|ATATCCATCAATGAATTTTATATTTTGATGCATTTCAAAGTAAATTGGTTTCAATAAAATGATTCAAA
                    >chr18:63162978-63163325 - E=4e-197
TGTGTTCTAAGGTCTTTCATAGTTCACATTGGTTTCCAGCTTACATTACTTCTATGATGCAATATTTCAGAATATCATCCAAATTAATA

GAGTTTACTCAGACATTTGTCTCAGTCTTGGACTCTGACTATTGTCCCACTAACATTGTGAGGCTGCCATAACTGCAGTGCTTAACCTT

TGGCTCTGAGCTTTTCAAGTGCCTGCTCTGAGATGTATCATGCTTCTAAGAGGAAACTGACCTTGAATACTGGGCTCACTCCCTATACC

TCCATCCTACTC
```
